# Notch-independent RBPJ controls angiogenesis in the adult heart

**DOI:** 10.1038/ncomms12088

**Published:** 2016-06-30

**Authors:** Ramón Díaz-Trelles, Maria Cecilia Scimia, Paul Bushway, Danh Tran, Anna Monosov, Edward Monosov, Kirk Peterson, Stacey Rentschler, Pedro Cabrales, Pilar Ruiz-Lozano, Mark Mercola

**Affiliations:** 1 Sanford Burnham Prebys Medical Discovery Institute, La Jolla, California 92037, USA; 2 Department of Bioengineering, Jacobs School of Engineering, University of California, San Diego, La Jolla, California 92093 USA; 3 Division of Cardiology, Department of Medicine, University of California, San Diego, La Jolla, California 92093 USA; 4 Departments of Medicine, Developmental Biology and Biomedical Engineering, Washington University, St Louis, Missouri 63110 USA; 5 Department of Pediatrics, Stanford University, Stanford, California 94305 USA; 6 Stanford Cardiovascular Institute and Department of Medicine, Stanford University, Stanford, California 94305, USA

## Abstract

Increasing angiogenesis has long been considered a therapeutic target for improving heart function after injury such as acute myocardial infarction. However, gene, protein and cell therapies to increase microvascularization have not been successful, most likely because the studies failed to achieve regulated and concerted expression of pro-angiogenic and angiostatic factors needed to produce functional microvasculature. Here, we report that the transcription factor RBPJ is a homoeostatic repressor of multiple pro-angiogenic and angiostatic factor genes in cardiomyocytes. RBPJ controls angiogenic factor gene expression independently of Notch by antagonizing the activity of hypoxia-inducible factors (HIFs). In contrast to previous strategies, the cardiomyocyte-specific deletion of *Rbpj* increased microvascularization of the heart without adversely affecting cardiac structure or function even into old age. Furthermore, the loss of RBPJ in cardiomyocytes increased hypoxia tolerance, improved heart function and decreased pathological remodelling after myocardial infarction, suggesting that inhibiting RBPJ might be therapeutic for ischaemic injury.

## Abstract (web summary)

Heart function after injury improves upon formation of new blood vessels. Here, the authors show that ablating a transcription factor RBPJ in the murine heart increases vascularization and maintains cardiac function after injury by increasing responsiveness to hypoxia, suggesting a new approach to treat heart injury.

## Graphical abstract

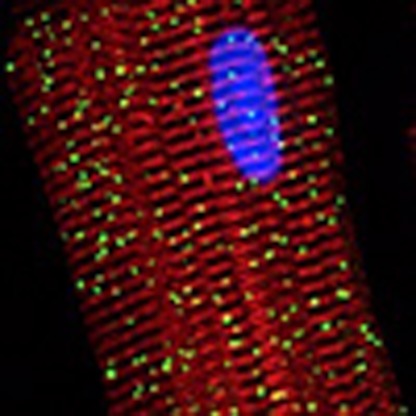

Angiogenesis in the adult heart is tightly regulated in order to maintain cardiac function in response to workload^1^. The mechanisms that coordinate angiogenesis and workload are poorly understood, but involve the regulated secretion of angiogenic factors from cardiomyocytes^2^,^3^.

Increasing angiogenesis is of therapeutic interest to treat chronic myocardial ischaemia,^4^,^5^ as well as acutely after myocardial infarction since many patients have inadequate perfusion despite restoration of coronary artery blood flow^6^. Despite pre-clinical proof of concept, clinical trials of single factor, gene and cell-based therapies have provided inconsistent results, and identifying a definitive means of inducing clinically useful therapeutic angiogenesis remains elusive^4^,^5^,^6^. A challenge in achieving therapeutic angiogenesis is that the formation of functional vessels involves the coordinated activities of numerous factors, some of which promote endothelial cell proliferation and vessel sprouting (for example, vascular endothelial growth factor (VEGF) and fibroblast growth factor (FGF)), while others drive vessel remodelling and maturation (for example, angiopoietins)^7^.

Since pressure overload increases myocardial angiogenesis and activates Notch signalling^8^,^9^, we hypothesized that Notch and its transcriptional effector RBPJ (recombination signal binding protein for immunoglobulin kappa J region) might function in cardiomyocytes as a high level modulator of myocardial angiogenesis. RBPJ is ubiquitously expressed, and can either activate genes by forming a complex with the Notch intracellular domain (NICD) when Notch is active^10^, or silence an overlapping but non-identical set of genes by recruiting co-repressors in the absence of Notch signalling^11^,^12^,^13^.

Our studies revealed that RBPJ represses the production of pro-angiogenic and angiostatic factors in cardiomyocytes of the unstressed heart, such that the adult cardiomyocyte (ACM)-specific deletion of *rbpj* increases microvessel density, confers physiological tolerance to hypoxia and protects the heart from ischaemic injury. Contrary to expectations, activation of Notch itself did not induce an angiogenic programme. We present evidence that RBPJ controls angiogenic factor gene expression by direct binding and inhibition of HIF1α and 2α. Thus, RBPJ has a novel Notch-independent role as a repressor of hypoxia responsiveness and myocardial angiogenesis.

## Results

### RBPJ acts as a transcriptional repressor in healthy ACM

**Figure 1:**
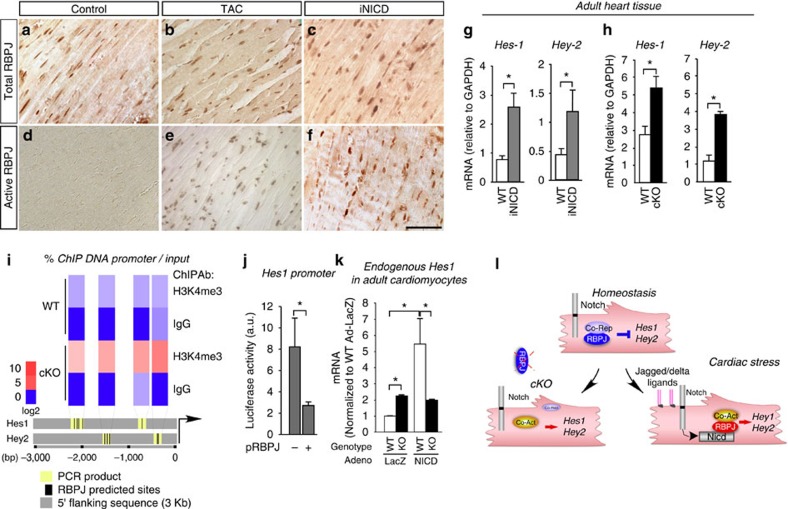
Cardiac stress switches RBPJ from transcriptional repression to activation. (**a**–**f**) Histological sections of ventricular myocardium from WT mice from sham (**a**,**d**) and TAC (**b**,**e**) operated mice, and a cardiomyocyte-specific doxycycline-iNICD mouse (**c**,**f**) immunostained for total RBPJ (**a**–**c**) and RBPJ in its transcriptional activating complex(brown) (**d**–**f**). TAC and iNICD upregulate nuclear-localized RBPJ. Scale bar, 50 μm. (**g**,**h**) qRT-PCR of Notch target genes (*Hes1*, *Hey2*) in ventricular tissue from iNICD (**g**) and RBPJ cKO (**h**) with their respective WT controls showing upregulation of target genes in both cases. Error bars indicate SEM *n*=4 mice for each condition. mRNA levels were normalized to *Gapdh* levels. Asterisk, *P*<0.05. (**i**) Heatmap representation of ChIP analysis of H3K4me^3^ in the endogenous *Hes1* and *Hey2* loci of WT and cKO ventricular myocardium. PCR primers span RBPJ consensus binding motifs (see inset below heatmap). Specific H3K4me^3^ and IgG PCR product levels were normalized to input DNA (see Methods section), revealing upregulated levels (red tones) of H3K4me^3^ modified chromatin in the cKO relative to WT. (**j**) Activity of transfected RBPJ on a *Hes1* promoter-luciferase construct in HEK 293 T cells. Error bars indicate SEM *n*=5 per condition. Asterisk, *P*<0.05. (**k**) Activity of endogenous *Hes1* expression (qRT-PCR) in isolated ACM, 48 h after adenoviral infection to overexpress NICD compared to inert gene (LacZ) control. Error bars indicate SEM *n*=4 mice for each condition. mRNA levels were normalized to β*-actin* levels and plotted relative to WT Ad-LacZ condition. Asterisk, *P*<0.05. (**l**) Model showing that RBPJ toggles between transcriptional repression (basally) and activation in response to Notch activation. Loss of RBPJ (cKO) de-represses target genes.

We first investigated the location and activity of RBPJ in the mouse adult heart. Nuclear-localized and faint cytoplasmic RBPJ is readily detected in the adult heart, both in cardiomyocytes and non-myocytes, and its level does not change substantially following either chronic pressure overload by thoracic aortic constriction (TAC) or myocardial infarction (Fig. 1a,b; Supplementary Fig. 1). In contrast, RBPJ was nearly undetectable basally by immunostaining with the monoclonal antibody T6709 (ref. 14) (Fig. 1d), but was strikingly evident in response to a doxycyclin-inducible, transgenic NICD (iNICD)^15^ (Fig. 1c,f) and after TAC or myocardial infarction (MI) (Fig. 1e; Supplementary Fig. 1c–f) coinciding with elevated expression of Notch ligands (Supplementary Fig. 2a–d). T6709-detection correlates with Notch activation^14^, suggesting that RBPJ in the heart might repress gene expression basally and mediate Notch signalling after MI and pressure overload.

To investigate the potential function of RBPJ in the heart, we deleted *Rbpj* in ACM with *Myl2*-Cre (cKO). cKO mice had normal viability consistent with post-natal deletion (Supplementary Table 1; Supplementary Fig. 3a,b). Deletion increased expression of the endogenous Notch/RBPJ target genes *Hes1* and *Hey2* identically to the effect of inducing Notch activation (iNICD) in unstressed hearts (Fig. 1g,h), and increased the content of activating tri-methylated (K4) histone-H3 (H3K4me^3^) in a proximal region of the endogenous *Hes1* and *Hey2* promoter containing RBPJ-binding motifs (Fig. 1i). *In vitro*, RBPJ overexpression repressed a *Hes1* promoter-luciferase reporter (Fig. 1j). Finally, *Hes1* messenger RNA (mRNA) content was higher in isolated cKO relative to wild-type (WT) cardiomyocytes, and could be induced by adenoviral delivery of NICD in an RBPJ-dependent manner (Fig. 1k). These data indicate that RBPJ acts as a transcription repressor basally in cardiomyocytes, but is converted to an activator upon pressure overload or MI (Fig. 1l).

### Lack of RBPJ induces angiogenesis in the heart

**Figure 2:**
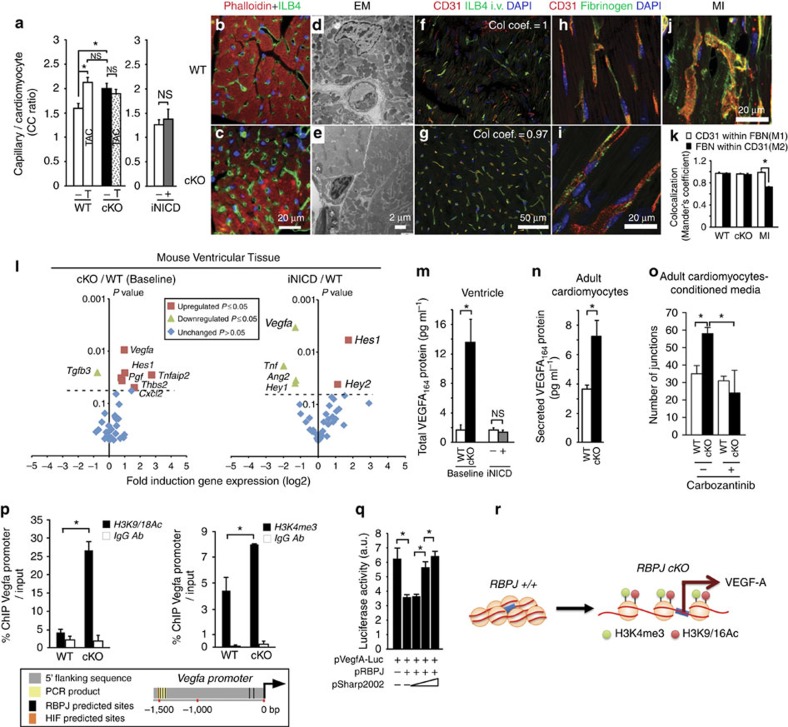
Cardiomyocyte-specific knockout of RBPJ increases myocardial angiogenesis. (**a**–**c**) Microvessel density quantification (**a**) on histological sections (**b**,**c**). Experimental genotypes are cKO (*Myl*2^Cre/+^, *Rbpj*^flox/flox^) and WT (*Myl*2^+/+^, *Rbpj*^flox/flox^) sham (−) or TAC (T), and iNICD (*Myh6*^rtTA^, *tetO-NICD*) with (+) or without (−) doxycycline (DOX). *n*=4, mice for all conditions except WT/+TAC were *n*=3. Scale bar, 20 μm. (**d**,**e**) Electron microscopy of WT (**d**) and cKO (**e**) capillaries at 48 months. Scale bar, 2 μm (**f**,**g**) Perfusion analysis by ILB4 and CD31 co-localization after FITC-ILB4 injection. *n*=3 WT, and 5 cKO mice. Scale bar, 50 μm. (**h**–**k**) Co-localization between fibrinogen (green) and CD31 (red) in WT (**h**), cKO (**i**) and control WT-infarcted heart (MI) reflecting capillary integrity. (**k**) *n*=3. Scale bar, 20 μm. (**l**) Gene expression analysis of 29 secreted angiogenic factors and 3 Notch target genes in ventricles from cKO, WT and doxycycline-induced iNICD mice by qRT-PCR normalized to *ActB*. Volcano plots portray the cKO to WT ratio or iNICD to WT ratio relative to *P*-value. Red and green points indicate statistically significant (*P*≤0.05, *n*=4) induction or repression of gene expression, respectively; blue points indicate no statistically significant difference. (**m**,**n**) VEGFA_165_ protein quantification by ELISA in WT, cKO and iNICD heart tissue (**m**) and conditioned media from WT and cKO-cultured ACM *n*=4 (**n**). (**o**) Conditioned media from WT and cKO ACM were applied to HMEC1 cells and network formation quantified incubation in the absence or presence of the VEGF receptor inhibitor carbozantinib (2 μM). *n*=3. (**p**) Histone marks in the endogenous *Vegfa* locus. ChIP with control IgG (white bars) and anti-H3K9/18Ac or anti-H3K4me^3^ (black bars) from isolated ACM was amplified using primers spanning RBPJ and HIF consensus binding motifs (schematic) and results normalized to input DNA. *n*=3. (**q**) Repression of *Vegfa* promoter-luciferase (50 ng) activity by RBPJ (1 ng) and reversion by co-transfection with escalating doses (0.5, 1 and 10 ng) of the SHARP RBPJ-binding domain (2002-3664) (ref. 18). *n*=3. (**r**) Loss of RBPJ leads to acquisition of active chromatin modifications and induction of *Vegfa* transcription. Data are mean±SEM *P<0.05; ns, not significant; *n*, biological replicates.

Next, we evaluated the consequences of *rbpj* deletion. cKO hearts showed no evidence of cardiomyocyte pathology (see Supplementary Fig. 4 for a detailed phenotypic characterization), and responded normally to pressure overload for all parameters analyzed, including cardiac function (Supplementary Table 2) and hypertrophy (Supplementary Fig 5). However, the loss of RBPJ significantly increased the number of capillaries relative to cardiomyocytes (CC ratio) from 1.6±0.27 in WT hearts to 2.1±0.28 in cKO hearts (Fig. 2a–c). cKO capillaries were morphologically normal even into old age (20 months, Fig. 2d,e), and were perfused by ILB4 lectin to the same degree as WT capillaries (Fig. 2f,g). Fibrinogen was not detected outside the vessel walls (Fig. 2h–k), indicating that the capillaries were not leaky, in contrast to capillaries following myocardial infarction as a control (Fig. 2j,k), or following chronic hypoxia-inducible factor (HIF) stabilization^16^,^17^. Thus, deletion of *rbpj* in cardiomyocytes increased the density of functional capillaries without inducing cardiac pathology.

Capillary density normally increases in response to chronically elevated blood pressure. In WT mice, 2 weeks of TAC significantly increased the CC ratio to 2.2±0.33. Myocardial microvascularization did not increase after TAC in the cKO-RBPJ mice beyond the already elevated basal level (1.9±0.17 CC ratio, statistically indistinguishable from the basal level). One possible explanation is that TAC signals through Notch/RBPJ to promote angiogenesis. Contrary to our initial hypothesis, doxycycline-activated iNICD did not affect the CC ratio (Fig. 2a), suggesting that TAC-induced microvascularization does not involve Notch.

To probe the mechanism responsible for the increased microvascularization, we profiled the expression of mRNAs encoding 29 secreted angiogenic factors. Five were upregulated in the cKO relative to WT, including factors involved in sprouting and endothelial cell migration (for example, VEGF-A and PGF), or stabilization (THBS2), and just a single factor (TGFβ3) was downregulated (Fig. 2l). iNICD did not upregulate any angiogenic factor mRNAs (but downregulated *Vegfa*, *Tnf* and *Ang2*) despite upregulating the canonical Notch/RBPJ target genes *Hes1* and *Hey2* (Fig. 1j). Although consistent with no effect of iNicd on the CC ratio, the result was puzzling since the angiogenic factor genes behaved differently than canonical Notch/RBPJ target genes *Hes1* and *Hey2*.

We investigated the divergent effects of the cKO and iNICD genotypes on angiogenic factor expression using *Vegfa* as a paradigm. Consistent with a Notch-independent role, VEGF-A protein levels were elevated in cKO, but not iNICD, heart tissue relative to WT controls (Fig. 2m,n; Supplementary Fig. 6). Culture media conditioned by cKO isolated ACM showed an increased ability to form microvascular endothelial tubes *in vitro* relative to WT-conditioned media, and this increased activity was blocked by the VEGF receptor inhibitor (carbozantinib) (Fig. 2o). Taken together, the *in vitro* and *in vivo* data support the model that RBPJ inhibits VEGF-A dependent angiogenesis. Chromatin immunoprecipitation revealed increased content of histone-H3 modifications (K9/K18Ac^2^ and K4me^3^) in the endogenous *Vegfa* promoter in a region ∼1.5 kb upstream of the start site of transcription that contains both RBPJ and HIF consensus motifs (Fig. 2p; Supplementary Table 3). These epigenetic marks indicate gene activation in the cKO cardiomyocytes. Activation was reversed by the overexpression of RBPJ, and co-transfection with a truncated-form of SHARP that physically sequesters RBPJ^18^ completely eliminated repression (Fig. 2q). Thus, RBPJ represses *Vegfa* gene expression under unstressed conditions, and deleting RBPJ leads to *Vegfa* gene activation (Fig. 2r).

### Cardioprotection and hypoxia tolerance in *rbpj* cKO hearts

**Figure 3:**
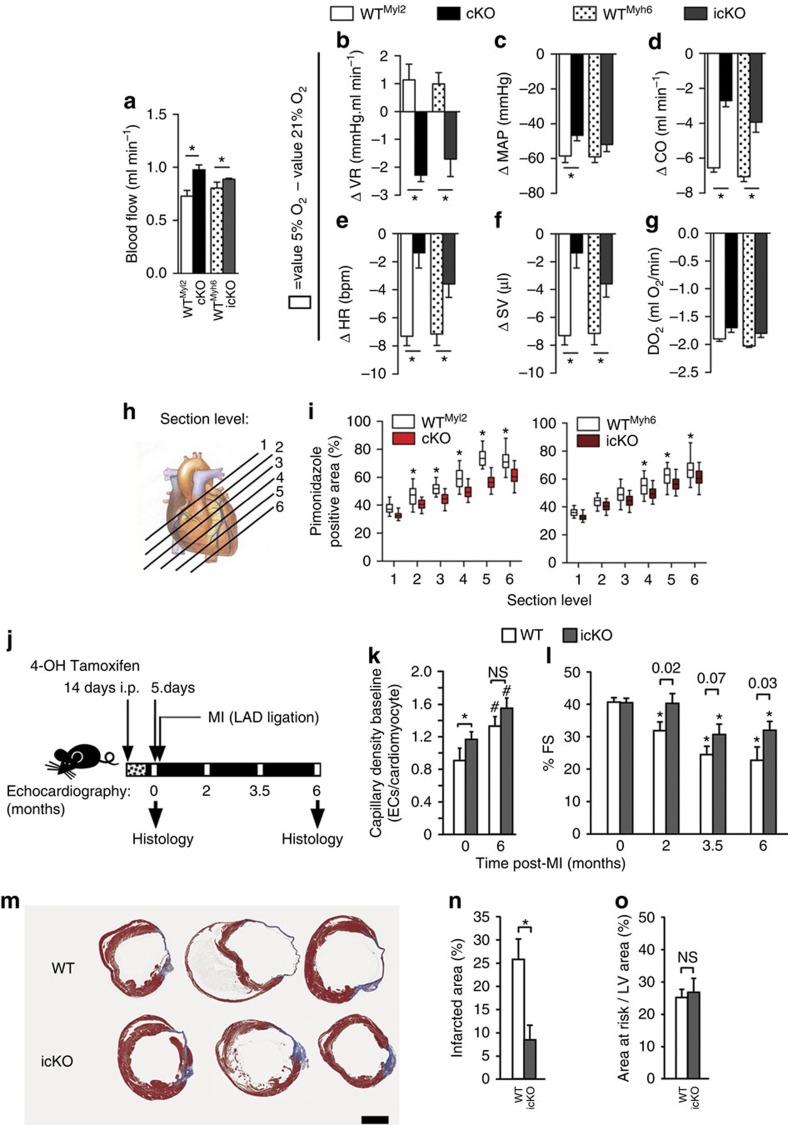
Loss of RBPJ induces hypoxia tolerance and cardioprotection after MI. (**a**–**g**) Hemodynamic parameters. Blood flow at baseline (**a**) and hemodynamics upon hypoxemic challenge (**b**–**g**), plotted as value at 5 versus 21% inhaled O_2_ in cKO and icKO (*Myh6*^mER-Cre-mER/+^, *Rbpj*^*fl*/fl^) mouse lines relative to their respective genotype controls (WT^Myl2^ or WT^Myh6^). Parameters were VR (**b**), MAP (**c**), CO (**d**), HR (**e**), SV (**f**), and O_2_ delivery (DO_2_) (**g**). *n*=4; *P<0.05 (ANOVA). (**h**,**i**) Tissue hypoxia quantified by pimonidazole detection in parallel histological sections, diagrammed (**h**). Apical sections (towards 6) had significantly smaller hypoxic regions in the cKO (left graph) and icKO (lower graph) hearts compared to their WT controls (**j**). Box represents mean and 25th and 75th percentiles; whiskers 5th and 95th percentiles. *n*=4 mice (**j**) Experimental protocol for MI. 4-OH tamoxifen was injected to induce recombination of the icKO at daily intervals during a 14-day window ending 5 days prior to LAD ligation (MI), followed by serial echocardiography and histological analysis at endpoint. (**k**) Microvessel density quantified 6 months after MI on histological sections. Capillary density was examined in the IVS, rather than the anterior wall, since the IVS was not damaged by the infarction. *n*=3, 4, 6, 6 for WT, icKO, WT+MI, icKO+MI, respectively. (**l**) LV fractional shortening (%FS) (*n*=9 for each genotype and timepoint). *P* value between genotypes listed. *P<0.05 respect to baseline same genotype. (**m**,**n**) Cross-sectional views (**m**) of WT and icKO hearts 6 months after MI and stained with Masson's trichrome at the level of the papillary muscles. Quantification of the scar area (**n**) in infarcted myocardium (blue histological stain) expressed as a fraction of LV circumference from 3 parallel cross-sections spaced 700 μm apart (*n*=6 WT and 7 icKO; see Methods section). Scale bar, 1 mm. (**o**) LV hypo-perfusion (Area At Risk, AAR) determined by intravenous injection of FITC-ILB4 2-days post-MI and quantified on histological sections at the level of papillary muscles (*n*=3 mice each genotype, see Methods section). Note that both genotypes showed comparably sized areas of hypo-perfusion. Data are means± SEM. *P<0.05; ns, not significant; *n*, biological replicates.

Given the increased CC ratio in the cKO heart, we evaluated the hemodynamic consequences deleting *Rbpj*. In addition to the cKO genotype, we generated a tamoxifen-inducible *Myh6*-Cre^19^ knockout (icKO; *Myh6*^mER-Cre-mER/+^, *Rbpj*^*f*l/fl^) to afford precise control over the timing of *Rbpj* deletion. Blood flow within left ventricular myocardium was greater in the icKO and cKO hearts than in WT controls (Fig. 3a). icKO and cKO mice (along with genotype-specific WT controls) were subjected to acute hypoxia by gradually decreasing ambient O_2_ from 21 to 5%. The different groups (cKO, icKO and their WT controls) had similar hemodynamic values under normoxic conditions (21% O_2_) (Supplementary Table 4). At 5% O_2_, vascular resistance (VR) increased as expected, but was significantly lower in cKO and icKO than WT mice (Fig. 3b), correlating with elevated mean arterial pressure (MAP) and cardiac output (CO) (Fig. 3c–f). Although not statistically significant, O_2_ delivery at baseline and 5% O_2_ was greater in the cKO and icKO relative to WT (Fig. 3g). Myocardial hypoxia (measured on histological section) in the cKO and icKO was consistently diminished by 10–20% relative to WT controls (Fig. 3h,i). Thus, loss of RBPJ in ACM correlated with increased myocardial oxygenation and CO under hypoxia.

Since stimulating angiogenesis has been considered a potential therapy for ischaemic disease^5^,^6^,^20^, we evaluated the response of icKO mice to MI by serial echocardiography and histology (Fig. 3j). The icKO hearts had increased CC ratio at baseline and preserved cardiac function (Fig. 3l) and morphology (Fig. 3m) relative to WT hearts post-MI (Fig. 3l,m), here measured in the interventricular septum (IVS) since the left ventricle (LV) free wall was injured by the MI. The extent of fibrotic scar formation was also diminished significantly, from 25±4.3% of the total LV area in WT to ∼8±3.0% in icKO hearts (Fig. 3n). The area of the myocardium that was hypo-perfused acutely (24 h) post-MI was identical in both genotypes, quantified by FITC-ILB4 perfusion (∼25% of total LV area) (Fig. 3o), and there was no effect on arterial arborization (Supplementary Fig. 4,k,k′), demonstrating that the immediate effect of left anterior descending (LAD) occlusion was the same in both genotypes. We conclude that deleting RBPJ improved tolerance to MI.

### RBPJ physically inhibits HIFs

**Figure 4:**
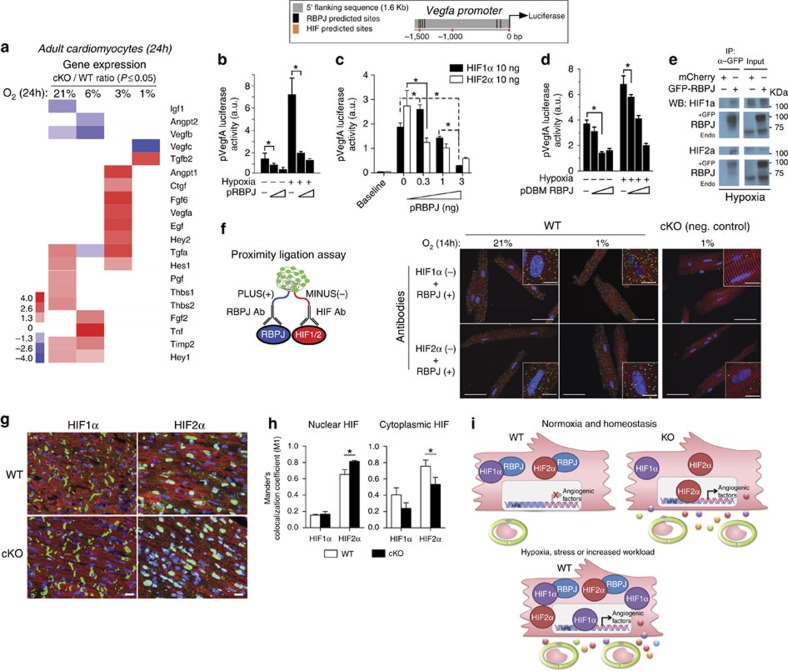
RBPJ suppresses myocardial angiogenesis by antagonizing HIFs. (**a**) The heatmap represents the ratio of cKO to WT gene expression for statistically significantly induced (red) or repressed (blue) genes (Mann–Whitney, *n*=4, *P*≤0.05; see Methods section) in isolated cardiomyocytes cultured for 24 h at the indicated O_2_ levels. White indicates no differences. Inset to left shows colour scale. See Supplementary Fig. 7 for complete dataset. (**b**–**d**) *Vegfa* promoter (see inset) driving luciferase (50 ng) was co-transfected with increasing amounts (1–5 ng) of an RBPJ expression plasmid (pRBPJ) and is activity tested after 24 h at baseline, hypoxia (1% O_2_) (**b**) or upon activation by HIF1α or HIF2α expression plasmids (10 ng) (**c**) in HEK 293 T cells. Increasing concentrations of the DNA-binding mutant (DBM) of RBPJ, pDBM-RBPJ also inhibited *Vegfa* promoter basally and as induced by 24 h of hypoxia (1% O_2_) (**d**). *n*=3 on (**b**) and 5 on (**c**,**d**) biological replicates. (**e**) Co-IP of eGFP-tagged RBPJ with endogenous HIF-1α and HIF-2α under hypoxic (2% O_2_) conditions. (**f**) Visualization of RBPJ interaction with HIF-1α and HIF-2α by *in situ* proximity ligation assay (PLA). Isolated ACM cultured for 14 h under hypoxia (1% O_2_) or normoxia (21% O_2_) were incubated with RBPJ probe (conjugated with PLUS oligonucleotide) and HIF1α or HIF2α probes (conjugated with MINUS oligonucleotide). Positive interactions in WT cardiomyocytes (green fluorescence) are seen in the cytoplasm (α−actinin^+^, red) and nuclei (DAPI, blue). cKO ACM were used as a negative control to show PLA signal specificity. Pictures are representative of at least three experiments. Scale bars, 50 μm, insets 10 μm. (**g**,**h**) HIF-1α and HIF-2α detected (both in green) in cKO and WT heart sections, co-stained with α-actinin (red) and DAPI (blue) (**g**). Manders co-localization coefficient M1 for HIF1α or HIF2 α within nuclei (DAPI) (left graph, **h**) or cytoplasm (α-actinin^+^). For M2 of DAPI and α-actinin within HIF1 α and HIF2 α, see Supplementary Fig. 9). *n*=3 and 4 hearts for WT and cKO mice. Scale bar, 20 μm. (**i**) Paracrine regulation of myocardial angiogenesis. RBPJ antagonizes HIFs to homeostatically repress angiogenesis. HIF stabilization in response to decreased O_2_ increases angiogenesis. All data are means±SEM. *P<0.05; ns, not significant; *n*, biological replicates.

The preceding showed that deleting RBPJ increases angiogenic factor gene expression and microvessel density, correlating with improved cardiac function after MI. We then investigated the mechanism responsible for the puzzling finding that RBPJ represses angiogenic factor genes basally, but that Notch/RBPJ does not induce them. Since hypoxia is a major regulator of angiogenesis^2^,^6^,^21^, we examined whether hypoxia responsiveness is altered by the cKO. Exposing isolated ACM to severe hypoxia (1% O_2_) upregulated 13 and downregulated 4 of the 29 secreted angiogenic factor genes profiled relative to normoxia (21% O_2_) (Supplementary Fig. 7). The response at 1% O_2_ was nearly identical in WT and cKO genotypes. However, plotting the ratio of gene expression change in cKO relative to WT revealed that the loss of RBPJ sensitized angiogenic genes to induction by moderate hypoxia (6–3% O_2_) (Fig. 4a).

Given that the −1.5 kb region of the *Vegfa* promoter contains both RBPJ and HIF consensus binding sites (Fig. 4b, inset; Supplementary Table 3), we hypothesized that RBPJ might interact with HIF to mediate repression. In a luciferase assay, RBPJ repressed induction of the *Vegfa* promoter by hypoxia (Fig. 4b) as well as by HIF1α or Hif2α (Fig. 4c) in a dose-dependent manner.

Repression occurred identically with a mutant RBPJ containing four mutations that block binding to recognition sites in DNA^22^ (Fig. 4d), suggesting that RBPJ does not need to interact with DNA in order to inhibit HIF.

Overexpressed GFP-tagged RBPJ in HEK293T cells under hypoxia was co-immunoprecipitated with endogenous HIF1α and HIF2α (Fig. 4e), suggesting a physical interaction between the proteins. Furthermore, a proximity ligation assay (PLA, Fig. 4f) revealed that RBPJ co-localizes with both HIF1α and HIF2α in ACM under normal and hypoxic conditions *in vitro*. Cytoplasmic and nuclear co-localization was apparent, consistent with the distribution of HIF and RBPJ in both compartments (Supplementary Fig. 8) and the ability of the RBPJ DNA-binding mutant to antagonize hypoxia and HIF-dependent *Vegfa* transcription. To control for non-specific interactions, no fluorescent signal was detected in cKO cardiomyocytes (Fig. 4f). Both HIF1α and HIF2α are detectable in WT and cKO mouse hearts (Fig. 4g), although HIF2α is more prominent in nuclei (65% (HIF2α) versus 15% (HIF1α) in WT cells) and increases further in the cKO (80% (HIF2α) versus 15% (HIF1α) in cKO cells) (Fig. 4h; Supplementary Fig. 9). These data indicate that RBPJ physically interacts with HIF proteins basally, and that repression is overcome by HIF stabilization under hypoxia.

## Discussion

In summary, RBPJ regulates a paracrine circuit for myocardial angiogenesis (Fig. 4i). RBPJ exerts control over microvessel density by acting within cardiomyocytes to antagonize HIF1α and HIF2α; therefore, desensitizing the angiogenic response to hypoxia. Deleting RBPJ, therefore, increases sensitivity to hypoxia. Attenuating RBPJ in cardiomyocytes is remarkable, in that it benignly increases myocardial vascularization, increases cardiac perfusion and output under hypoxia and preserves cardiac function following MI. There are relatively few instances of RBPJ having a repressive role in mammals^23^,^24^,^25^, and most studies have focused primarily on its function as the effector of Notch signalling, which is itself cardioprotective following ischaemic injury^8^,^9^,^26^. The effect of RBPJ deletion resembles chronic adaptation of the heart to hypoxia^27^ suggesting that attenuating the suppressive function of RBPJ might be therapeutic in the setting of ischaemic injury or chronic overload.

## Methods

All animal handling and care followed the NIH Guide for Care and Use of Laboratory Animals. The experimental protocols were approved by Institutional Animal Care and Use Committees of either the Sanford-Burnham-Prebys Medical Discovery Institute, the University of California San Diego or Washington University. Unless otherwise noted, approximately equal numbers of 8–16-week-old male and female mice were used in all studies.

### Genetic ablation of RBPJ in adult mouse hearts

To obtain cardiomyocyte-specific deletion of RBPJ in adult mouse hearts we created the following mouse lines. *Myl*2^Cre/+^ transgenic mice (Black Swiss) were crossed to *Rbpj*^flox/flox^ mice (C57BL/6)^28^ to yield cKO (*Myl*2^Cre/+^, *Rbpj*^flox/flox^) and phenotypically WT (*Myl*2^+/+^, *Rbpj*^flox/flox^) littermates. *Myh6*^cre/Esr1^ transgenic mice^19^ were also crossed to *Rbpj*^flox/flox^ mice to obtain cKO *Myh6*^mER-Cre-mER/+^, *Rbpj*^*f*l/fl^ and phenotypically WT *Myh6*^mER-Cre-mER/+^, *Rbpj*^+/+^.

### Tamoxifen induction of recombination

4-OH tamoxifen (Sigma) prepared as 5 mg ml^−1^ in corn oil was injected (0.1 ml per mouse) intraperitoneally at daily intervals during a 14-day period that ended 5 days before LAD ligation, as described^29^, or 30 days before physiology tests.

### Genetic activation of Notch receptor in adult mouse hearts

*αMHCrtTA*; *tetO-NICD* mouse line was previously generated^15^ to activate Notch signalling by inducing NICD specifically in ACM. NICD was induced with doxycycline chow (BioServ 200 mg kg^−1^) during 2 weeks. *αMHCrtTA* and *tetO-NICD* littermates fed doxycycline were used for comparison in all conditional gene expression experiments unless otherwise noted. Littermate controls were used for all experiments. Animal protocols involving these mice were approved by the Animal Studies Committee at Washington University.

### Statistical analysis

Data are shown as means ±standard error of the mean (SEM) unless otherwise specified. *P* values were calculated by the Mann–Whitney, the Student's t-test or the one-way ANOVA, for samples *n*=3 and *n*≥4, respectively, from independent biological replicates and the two-way ANOVA for physiology experiments.

### Heatmap analysis

Quantitative reverse transcription polymerase chain reaction (qRT-PCR) data was internally normalized for each genotype and condition to *ActB* levels. The amount of complimentery DNA (cDNA) used was titrated and verified to yield cycle threshold (CT) values in the linear response range. All conditions where normalized to the values of WT cells at 21% oxygen levels. Resulting data was hierarchically clustered using Cluster 3.0 using the complete linkage cluster method. Java TreeView software generated the colour map.

### Inmunocytochemistry and histochemistry

Hearts were arrested in diastole by perfusion with 300 mM KCl solution before fixation in 4% paraformaldehyde in PBS. They were then processed for either paraffin embedding or fresh frozen directly in optimum cutting temperature compound (OCT) directly after collection. Briefly, paraffin sections (7 μm thick) were de-paraffinized, rehydrated and heat-treated in Degloacker buffer pH 9 for antigen retrieval. After several PBS washes, sections were blocked with 5% BSA in PBS and incubated overnight at 4 °C with the rat primary anti-RBPJ antibody (generously provided by Professor T. Honjo, Kyoto University^30^) (1:50), rabbit monoclonal RBPJ (CST) (1:300 or 1:2,000), rabbit anti-Jagged-1 (SCBT) (1:300), rabbit anti-Delta-Like 4 (SCBT) (1:100), mouse α-actinin (Sigma) (1:500), rabbit HIF1alpha (1:100 or 1:1,000 for TSA) and HIF2 alpha (1:100 or 1:1,000 for TSA) (Nobus). After PBS washing, sections were incubated with secondary antibodies. Either an HRP-conjugated anti-rat IgG, HRP-conjugated anti-rabbit IgG, alexa fluor-conjugated (488 or 586) goat anti-mouse or goat anti-rabbit IgG were applied for one hour at room temperature. HRP was detected by DAB or by the thyramide signal amplification system (Molecular Probes) for Hif1 and HIF2 alpha. To visualize microvessels, sections were incubated with FITC-conjugated *Lycopersicon esculentum* agglutinin (FITC-LEA, Sigma) (1:1,000) and Rhodamine-phalloidin (Invitrogen) (1:1,000) for 2 h in PBS with calcium and magnesium, and subsequently counterstained with DAPI. Sections were mounted and photographed on a Zeiss Axiophot microscope with a 40 × objective (N.A. 0.75) or on a Zeiss Imager.Z1 plus apotome with 40 × (N.A. 1.2w) objectives.

Hif1 and HIF2 alpha antibodies were validated on heart tissue from embryonic day E11.5 mouse where Hif1 and HIF2 alpha had been deleted. The genotype of the HIF1 and HIF2 conditional KO mice correspond to F*Gata5* Cre+ × *Hif1a* F/− and F*Gata5* Cre/+ × *Hif2a* F/−, respectively. The F*Gata5* Cre mice have direct Cre recombination in the epicardium as well as in regions of the myocardium^31^.

For frozen sections, tissue was fresh frozen in OCT. Histological sections were fixed in methanol/acetone (1:1) at −20 °C for 2 min and processed as described above, but the antigen retrieval step was omitted. Frozen sections were incubated with goat anti-fibrin/fibrinogen (GAM/Fbg/Bio; Nordic Immunology) (1:500), rat anti-mouse CD31 (BD-Pharmingen) (1:100), anti-alpha smooth muscle actin (Sigma) (1:500) primary antibodies and alexa fluor-conjugated (488 or 586) streptavidin or goat anti-rat or goat anti-mouse IgG (all 1:500). Biotin blocking was performed as described for the Streptavidin/Biotin blocking kit (Vector Labs) on those sections incubated with biotin-conjugated antibodies.

For histochemistry, hearts were fixed in 4% formaldehyde and processed for paraffin embedding. Sections were stained for Massons' trichrome (collagen stain), PAS (periodic acid acid-Schiff) (to detect glycogen) and Oil red-O (to detect lipid) following standard procedures (Sanford-Burnham-Prebys Histology Core Facility). Sections were mounted and photographed on a TE300 Nikon microscope with 40 × (N.A. 0.75) objective.

For plastic casting of coronary vascular trees, Microfil polymer (Flow Tech, Inc.) was perfused through the aorta of isolated hearts^2^. Mice had been previously injected with heparin and the heart washed with saline solution to remove blood. After polymer injection hearts were processed following manufacturer's instructions and imaged on a Nikon SMZ stereo-microscope.

### Electron microscopy

For transmitted electron microscopy, hearts were arrested in diastole by perfusion with 300 mM KCl, dissected and sliced in 1 mm thick sections and fixed in 2% pFA/2% glutaraldehyde in 0.1 M cacodylate buffer solution, pH 7.4. Slices were then washed and incubated in 1% osmium tetroxide in the same buffer, dehydrated in ethanol and embedded in Eponate 12 epoxy resin (Ted Pella Inc. C, Redding, CA, USA). Ultrathin sections were prepared on a Reichert-Jung Ultracut microtome and stained with uranyl acetate and lead citrate, and examined using a Hitachi H-600A electron microscope, at 75 kV. Digital images were acquired with 2K × 2K CCD cooled monochrome 12-bit camera L3C (SIA, Atlanta, GA, USA) using “Picture Frame” software (Optronics, Inc., Goleta, CA, USA). Images were processed with ImageJ (public domain software) to achieve a similarity in contrast and brightness of all images.

### Imaging quantification

Imaging quantification was done with ImageJ. Colocalization analysis was performed using a colocalization analysis plugin (http://www.uhnres.utoronto.ca/facilities/wcif/imagej/colour_analysis.htm) that generates Mander's coefficients^32^. Determination of distances on images (for example, Z-line and cardiomyocyte cross-sectional area measurements) were done by manually selecting the distances to be measured. For the analysis of the size of ACM *in vitro*, brightfield images were process on ImageJ as described (http://rsbweb.nih.gov/ij/docs/pdfs/examples.pdf) and quantified using the particle size analysis function.

### Intravital endothelial cell labelling

Mice were anesthesized by intraperitoneally (i.p.) ketamine (100 mg kg^−1^)-xylazine (2.5 mg kg^−1^) injection followed by injection with heparin (1,000 U kg^−1^) to reduce formation of thrombi. To label the endothelial cells intravitally, the anesthetized mice received a jugular injection of 0.1 ml FITC-conjugated Isolectin B4 (ILB4, Vector Lab). ILB4 was allowed to circulate for 15 min. Mice were then sacrificed and hearts collected in 4% paraformaldehyde, washed in PBS, cryopreserved in sucrose, and embedded in OCT and process for frozen sections.

### ACM preparation and culture

ACM were isolated from 3 to5-month-old mice by Langendorff perfusion method as described^33^. Before plating, cardiomyocytes were separated from non-cardiomyocytes by sedimentation. Two hours after plating, adenovirus was applied to the media at an MOI of ∼50 pfu per cell for 24 h. After changing media on the next day, cardiomyocytes were incubated in a hypoxia chamber (Biospherix XVivo, UK) under specified O_2_ tension, as indicated in the text, for 24 h before collection of samples for gene expression analyses or immunodetection.

### Co-immunoprecipitation and western blot

For HIF1a and HIF2a interaction with RBPJ, HEK 293 T (ATCC) cells were transfected with either mCherry (negative control) or RBPJ-GFP expression vectors and subjected to hypoxia (2% O_2_) overnight before total protein extraction. Total extracts were prepared by incubating cells at 4 °C in lysis buffer (PBS with 0.5% Triton-X 100). Lysates were centrifuged for 1 min at 12,000*g* to remove insoluble debris. Immunoprecipitation was preformed by incubating total extracts with anti-GFP antibody (1:100) preadsorbed to protein A/G-agarose (Santa Cruz Biotechnology) and rocking for 1 h at 4C. Beads were then washed 3 × with 1 ml of ice-cold lysis buffer prior to resuspension in 1 × Laemli buffer and subjected to western blot. Antibodies used for western blot were RBPSUH (D10A4) XP (Cell Signaling) (1:1,000), HIF-1 alpha (NB100-479) (1:500) and HIF-2 alpha (NB100-122) (1:500).

### *In situ* PLA and immunofluorescence

Isolated ACM seeded on coverslips were fixed on cold methanol for 10 min. After washing with PBS and permeabilization with 1% Triton X-100 cells were process for PLA or immunofluorescence.

For immunofluorescence, cardiomyocytes were incubated with blocking buffer (5% BSA, 0.1% Triton X-100) alone for 1 h and then with the primary antibodies overnight at 4 °C. Excess of antibody was removed by several PBS washes and alexa fluor-conjugated (488) secondary antibody with rhodamine-conjugated phalloidin (1:1,000) was applied with the blocking buffer for 1 h. After several PBS washes, coverslips were mounted on slides with Vectashield with DAPI (Vector Labs) and photographed.

The proximity ligation assay was performed on coverslips following the Duolink *In Situ* User Manual (sigma.com/duolink) and using Duolink *In Situ* Solutions (Sigma). First, we made PLA probes conjugating primary antibodies with a PLUS or MINUS oligonucleotide. We used the Duolink *In Situ* Probemaker PLUS (Sigma) for RBPJ antibody, RBPSUH (D10A4) XP (Cell Signaling) and MINUS for HIF-1 alpha (NB100-479) and HIF-2 alpha (NB100-122) antibodies. After blocking, cardiomyocytes were incubated with combinations of HIF-1 alpha probe (−) and RBPJ probe (+), or HIF-2 alpha probe (−) and RBPJ probe (+) overnight at 4 °C in a humidified chamber (all 1:250). After washing the antibody, we performed the ligation and amplification steps as described (sigma.com/duolink) using the Duolink *In Situ* Detecton Reagents Green (Sigma). After the final washes we incubated cells with rodamine-conjugated phalloiding for 10 min before mounting the coverslips on glass slides using Vectashield with DAPI (Vector Labs).

Images were taken on a Zeiss Imager.Z1 plus apotome with 40 × (N.A. 1.2w) objectives and processed with ImageJ (NIH, Rasband, W.S., ImageJ, U. S. National Institutes of Health, Bethesda, Maryland, USA, http://imagej.nih.gov/ij/, 1997–2014).

### Protein analysis

Mouse VEGF-A ELISA (Biosource, Invitrogen) was performed following manufacturer's instructions. ACM were isolated as described (see above) from WT sham and TAC mice and immediately collected on RIPA lysis buffer. Western blots were performed as described^34^. Briefly, samples were loaded and run on a 4–12% TGX-precast gels (Bio Rad) after being heated on 2 × Laemmli sample buffer with β-mercaptoethanol (Bio Rad) for 10 min at 70 °C. proteins were then transferred using the Trans-Blot Turbo Transfer System (Bio Rad) into PVDF membranes that were blocked with 5% Difco skin milk (BD Bioscience) before antibody incubations. Antibodies used were rat primary anti-RBPJ (1:500) antibody (generously provided by Professor T. Honjo, Kyoto University^30^), ANF (1:500) (Millipore, AB5490) and GAPDH (1:2,000) (Abcam, ab9485).

### Endothelial tube formation assay

A microvascular endothelial tube formation assay was performed as described^35^ to quantify microvessel formation in response to factors produced by the WT and cKO cardiomyocytes. Briefly, media conditioned by 18 h culture of mouse ACM was collected and centrifuged to remove dead cells and debris. The conditioned media was then applied to human microvascular endothelial (HMEC1) cells plated in 96-well plates previously coated with matrigel (7–8 mg ml^−1^, 45 μl well^−1^). The cultures were incubated for an additional 16 h either with or without the VEGF receptor inhibitor carbozantinib (Selleckchem). After the 16 h culture period, the angiogenic response was quantified automatically by determining the number of junctions formed using the macro ‘Angiogenesis Analyzer' by Gilles Carpentier (Gilles Carpentier. Contribution: Angiogenesis Analyzer, ImageJ News, 5 October 2012) for NIH Image J 1.47v Program.

### RNA extraction and qRT-PCR

RNA was extracted from cells and tissue using Trizol reagent (Invitrogen) and cDNA was made using the Qiagen Reverse Transcription kit. PCR with specific primers (see Supplementary Tables 5 and 6) was performed using the LightCycler FastStart Master SYBR Green Kit (Roche) in a LightCycler 2.0 (Roche). Gapdh and β-actin (Actb) were used as internal controls to normalize the data, as specified in the text.

For the angiogenic gene analysis, quantitative Real-Time PCR was performed for the different samples using the iQ SYBR Green Supermix (BioRad) and loaded on a ABI 7900HT. Values are expressed either as 2^ΔΔCt^, with ΔΔCt defined as the difference in crossing threshold (Ct) values between experimental and control samples as described^36^ or as Log_10_ 2^ΔCt^ using *Actb* (β-actin) as control gene.

### Luciferase assay

Luciferase reporter constructs were transfected into HEK293T cells on 24 or 384 well plates with Lipofectamine LTX. Overall, 24 h after transfection, cells were placed under hypoxia for 12–20 h as above. Samples were processed to assay luciferase activity using the Dual Luciferase Kit (Promega). Luciferase activity was normalized against total protein content quantified by Bradford colorimetric analysis (Pierce). pHes1 was a gift from Jan Jensen (Cleveland Clinic, Ohio); mouse 1.6 Kb pVegfa–Luc from Patricia D'Amore^37^ (Harvard Medical School, Boston, MA, USA), pSharp (2002-3664) DeltaB and pRbpj from Franz Oswald (Max-Planck-Institute of Immunobiology, Germany), HA-HIF1alpha-pcDNA3 and HA-HIF2alpha-pcDNA3 was a gift from William Kaelin (Addgene plasmid # 18949 and Addgene plasmid # 18950, respectively)^38^.

### Chromatin immunoprecipitation (ChIP) analysis

ChIP assays were performed using previously described methods^39^. Briefly, ACM from WT and RBPJ cKO mouse hearts were collected, fixed in 2% formaldehyde 12 min at room temperature, rinsed twice in ice-cold PBS, and lysed directly in sonication buffer (1% SDS, 10 mM EDTA, 50 mM Tris–HCl pH8.0 and protease inhibitors cocktail (Sigma)). The resulting cell suspension was sonicated in a waterbath sonicator (MiSonix Sonicator 3000) using the following programme: 30 s ON, 30 s OFF during10 min (Output Power: 4). The suspension was centrifuged (Eppendorff microfuge) and the cleared supernatant was incubated at 4 °C overnight with an antibody to di-acetylated Histone3 (K9, K18) (Millipore, 07-593) or to tri-methylated Histone3 (K4) (Abcam Ab8580) (both 1 μg/10^6^ cells). Normal rabbit IgG was the negative control (1 μg/10^6^ cells). Protein-DNA complexes were collected on Protein G beads (SIGMA). After rinsing in PBS, the final DNA samples were subjected to PCR. Primer sets specific for the mouse Vegfa, proximal promoters spanning Hif and RBPJ-binding sites were synthesized for this purpose (Supplementary Table 3). PCR conditions were 95 °C for 2 min, followed by 25−30 cycles at 95 °C for 30 s, 55−60 °C for 30 s and 72 °C for 30 s.

### Transverse aortic constriction (TAC)

Mice between 10 and 14 weeks of age were anesthetized with a ketamine (100 mg kg^−1^)-xylazine (2.5 mg kg^−1^) mixture administered i.p., and connected to a rodent ventilator after tracheal intubation^40^,^41^. The chest cavity was opened with scissors by a small incision at the level of the second intercostal space. After isolation of the aortic arch, a 7–0 silk suture was placed around the aorta and a 27-gauge needle. The needle was immediately removed to produce an aorta with a stenotic lumen. The chest cavity was then closed with one 6–0 nylon suture and all layers of muscle and skin closed with 6–0 continuous absorbable and nylon sutures, respectively. Sham-treated animals underwent surgery without the final tightening of the constrictive suture^40^,^41^. Gradients across the stenotic aorta were measured through echocardiographic analysis, and animals with pressure gradients less than 30 mmHg were not included in the study. Echocardiography was as described^42^. Both males and females were used and no significant gender difference was noted.

Hearts were collected at 14 days after the surgery, arrested in 300 mM KCl and fixed in 4% PFA, or fresh frozen in OCT for histological analysis or, frozen, and stored at −80 °C for RNA and protein extraction.

### MI mouse model

The protocol was essentially as described^43^. Briefly, an oblique 8-mm incision was made 2 mm away from the left sternal border toward the left armpit (1–2 mm below). The muscles were separated with care to avoid damaging blood vessels. The chest cavity was then opened at the 4th intercostal space taking care not to damage the lung. A chest retractor was inserted and opened gently to spread the wound to 8–10 mm in width. The pericardium was gently picked up with curved and straight forceps, pulled apart, and placed behind the arms of the retractor. The LAD coronary artery runs in the midst of the heart wall from underneath of the left atrium toward the apex and was typically visible by its pulsating bright red colour. In instances when the LAD artery could not be visualized, the left atrium was lifted so that the source of the LAD artery from the aorta is located. The position of the ligation depends on the volume of infarction desired. In this study, the LAD artery was ligated 1–2 mm below the tip of the left atrium in its normal position, which induces roughly 40–50% ischaemia of the LV. Once the site for ligation had been determined, the curved forceps were used to gently press on the artery a little below the subsequent ligation (to enhance the view of the artery and stabilize the heart). Next, with a tapered needle, a 7–0 silk ligature was passed underneath the LAD coronary artery. For easier and smoother passage the needle was bent in advance to make the curvature rounder. The ligature was then tied with three knots. Occlusion was confirmed by the change of colour (becoming pale) of the anterior wall of the LV. The chest cavity was then closed by bringing together the 4th and the 5th ribs with one or two 6–0 nylon sutures (with pressure applied to the chest wall to reduce the volume of free air). The muscles and skin were closed layer-by-layer with 6–0 absorbable and nylon sutures, respectively. The sham-operated mice underwent the same procedure without tying the suture but moving it behind the LAD artery. Both males and females were used and no significant gender difference was noted.

### Animal preparation for cardiac function measurements

Anaesthesia was induced by i.p. injection of sodium pentobarbital (50 mg kg^−1^) and core body temperature was maintained using a heating pad. Animal preparation included: (i) left femoral artery catheterization, (ii) tracheotomy (polyethylene-90 tube to facilitate spontaneous breathing), and (iii) left ventricle conductance catheter introduction through the right carotid artery. Animals were placed in the supine position on the heating pad, before the experimental procedure. Toe pinching test was performed at least every 5 min, and animals who responded received a small dose of sodium pentobarbital (10 mg kg^−1^) to prevent animal discomfort. Mice were suitable for the experiments if: (1) systemic parameters were within normal range, namely, heart rate (HR)>300  beat min^−1^, MAP>90 mmHg, and arterial O_2_ partial pressure (p_a_O_2_)>80 mmHg. Only males were used for these cardiac function measurements. Detailed protocols are described below.

### Hypoxia protocol for cardiac function

The anesthetized animals were placed on heating pad within seal acrylic box. As described before for awake animals, the O_2_ concentrations in the box was changed using compressed air, 15% O_2_ balance N_2_, 10% O_2_ balance N_2_; and 5% O_2_ balance N_2_. Each hypoxic step was maintained for 30 min, and measurements were completed 15 min after animal's acclimatization. Hypoxia was stopped if blood pressure dropped below 40 mmHg, and the animal was excluded from the study.

### Systemic parameters

MAP and HR were recorded continuously (MP 150, Biopac System; Santa Barbara, CA, USA).

### Cardiac function

The closed chest method was used to study cardiac function. Right common carotid artery was exposed to insert a 1.4F pressure-volume conductance catheter (PV catheter; SPR-839, Millar Instruments; Houston, TX, USA). The pressure-volume catheter was advanced passing through the aortic valve into the LV^44^. At baseline and the end of the experiment, a bolus of 15% hypertonic saline (10 μl) was intravenously injected to determine parallel volume 45. The pressure and volume signals were continuously acquired (MPVS300, Millar Instruments; Houston, TX, USA and PowerLab 8/30, AD Instruments; Colorado Springs, CO, USA). Left ventricular volume was measured continuously in conductance units (RVU; relative volume unit) and converted to actual blood volume (μl) at the end of the experiment.

### Cardiac pressure–volume indices

Cardiac function data were analyzed with PVAN software (Millar Instruments, Houston, TX, USA). All cardiac function parameters were averaged from 8 to 12 cardiac cycles at each time point. End systolic pressure (*P*_es_) was directly measured. Cardiac output (CO), stroke work (SW) and stroke volume (SV) were calculated. VR was calculated as using the MAP divided by the CO (VR=MAP/CO). Oxygen delivery (DO_2_) was calculated as the product of the total Hb by the O_2_ carrying capacity of saturated Hb (1.34 mLO_2_/g_Hb_) by the arterial blood O_2_ saturation and CO (DO_2_=[RBCHb × 1.34 × SA] × CO)^46^.

### Hypoxic areas

Hypoxia protocol for awake mice were followed as mentioned earlier. Vital organ hypoxic areas were measured using immunohistochemistry staining for pimonidazole bound to hypoxic zones. Mice received an infusion of the hypoxic marker Hypoxyprobe-1 (pimonidazole 40 mg kg^−1^) and 5 mg kg^−1^ Hoechst 33342 (Invitrogen Corp., Carlsbad, CA, USA) diluted in PBS (total volume, 100 μl), when they were exposed to 10% O_2_. Then, the mice received a second infusion of pimonidazole (40 mg kg^−1^) and Hoechst 33342 (5 mg kg^−1^) diluted in PBS (100 μl), when they were exposed 5% O_2_. Finally, mice were euthanized and their heart was removed. Tissues were fixed by immersion in formalin for 24 h at room temperature before transfer to 70% (v/v) ethanol. Lastly, tissues were cut into 100-μm thick sections.

### Pimonidazole immunohistochemistry

Sections were cleaned and rehydrated according to standard procedures. Monoclonal antibody directed against pimonidazole (included in the Hypoxyprobe-1 green kit) was used for immunohistochemical staining of the tissue sections. Fluorescence microscopy was performed using an Olympus BX51WI equipped with a high resolution digital CCD ORCA-285 (Hamamatsu Corp., Hamamatsu city, Japan) illuminated with a mercury burner and the appropriate fluorescent cubes (XF100-2 and XF02-2, Omega Optical, Brattleboro, VT). Images for pimonidazole antibody-stained areas and Hoechst were prepared using Wasabi Imaging Software (Hamamatsu Corp). The ratio of pixels stained for pimonidazole in each region, to the total cellular area of the image was calculated. Ten images were analyzed by section, and the results were pooled to determine the mean and standard deviation. To indicate the co-localization of pimonidazole and Hoechst in cell, images were superimposed.

### Data availability

All relevant data are included in the figures and/or Supplementary Data, or available from the authors.

## Additional information

**How to cite this article:** Díaz-Trelles, R. *et al.* Notch-independent RBPJ controls angiogenesis in the adult heart. *Nat. Commun.* 7:12088 doi: 10.1038/ncomms12088 (2016).

## Supplementary Material

Supplementary Information Supplementary Figures 1-10 and Supplementary Tables 1-6
