# Supplementary material for: Notch-independent RBPJ controls angiogenesis in the adult heart
Source: Nat Commun. 2016 Jun 30;7:12088. doi: 10.1038/ncomms12088 (PMC4931341; doi:10.1038/ncomms12088)
Supplement: Supplementary Information — Supplementary Figures 1-10 and Supplementary Tables 1-6 [file ncomms12088-s1.pdf]

SUPPLEMENTARY FIGURES AND LEGENDS

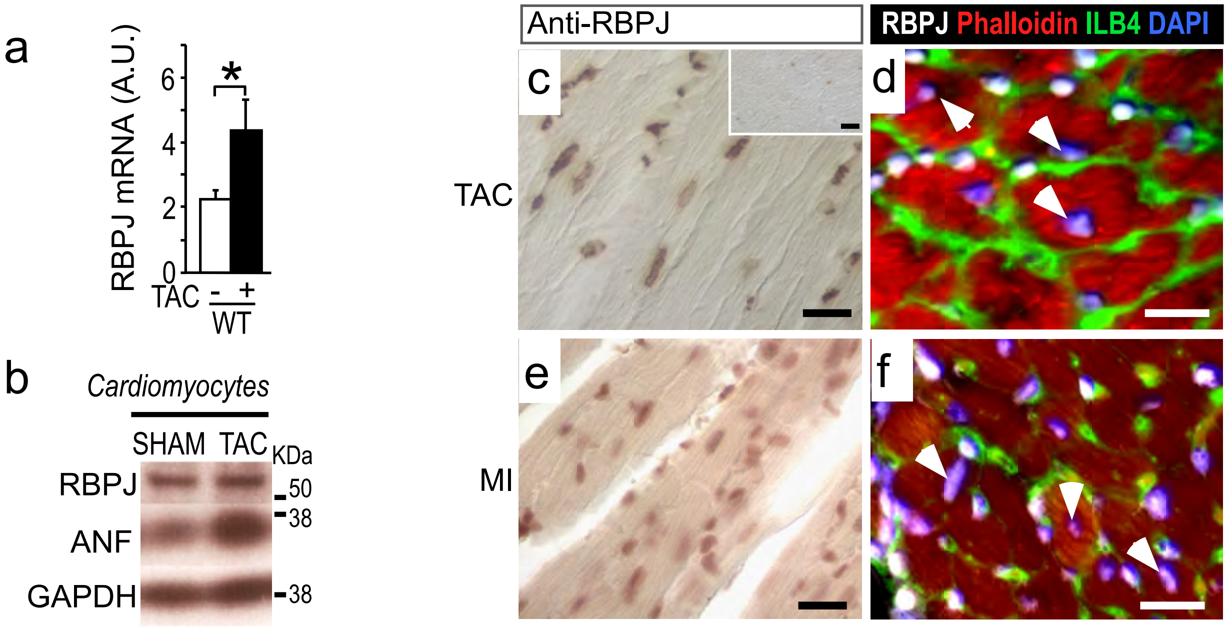

Supplementary Figure 1. Analysis of RBPJ in myocardium of WT mice.

a,b) RBPJ protein by mRNA Q-RT-PCR **(a)** and by Western blot **(b)** show a slight increase upon TAC in WT hearts. A.U., arbitrary units. Error bars in a indicate s.e.m. n=3 mice for each condition. mRNA levels were normalized to *Actb* (β-actin) levels (see Methods). Asterisk, $P<0.05$.

c-f) WT mice were subjected to TAC **(c,d)** MI **(e,f)** or unoperated (**inset**) and ventricular myocardium analyzed histologically at 14 days post-surgery. Brightfield micrographs showing immunostaining with T6709, an antibody that preferentially recognizes the activated form of RBPJ (brown) **(c,e)** and fluorescent micrographs showing T6709 (white) counterstained with anti-α-actinin (red) and FITC-LEA (green) **(d,f)** reveal upregulated nuclear-localized RBPJ after MI or TAC (arrowheads in **d** and **f** indicate cardiomyocyte nuclei). Scale bars **(c-f)**, 20 μm.

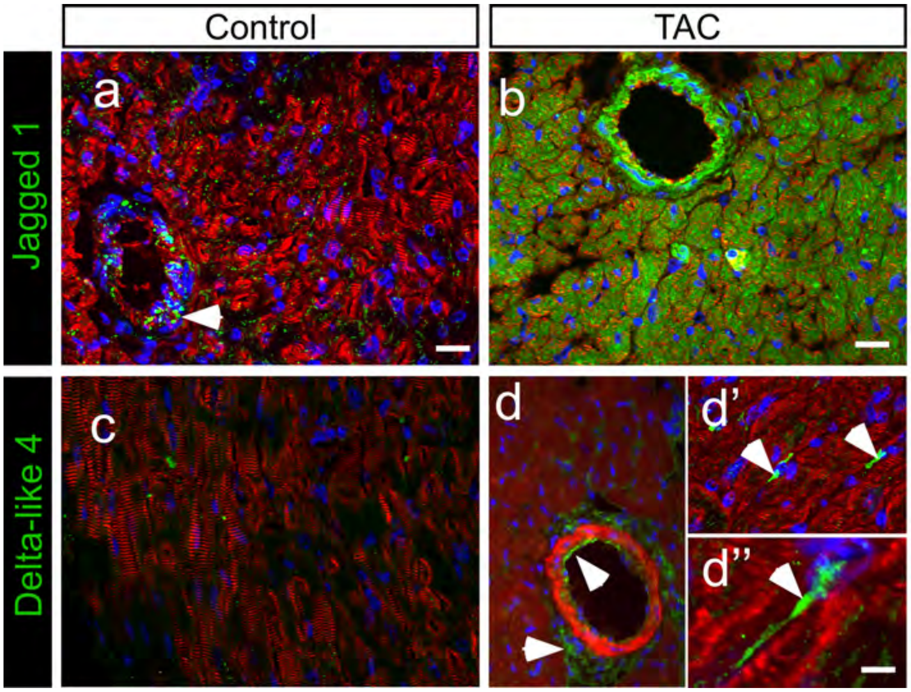

Supplementary Figure 2. Myocardial analysis of Notch ligands.

a-d) Histological immunostaining for Jagged-1 **(a,b)** and Delta-like-4 **(c,d,d',d'')** in left ventricular myocardium after 14 days of TAC or sham operation (control). Scale bars, 20 µm **(a-d')** and 5µm **(d'').** Arrowheads indicate staining in vascular endothelium

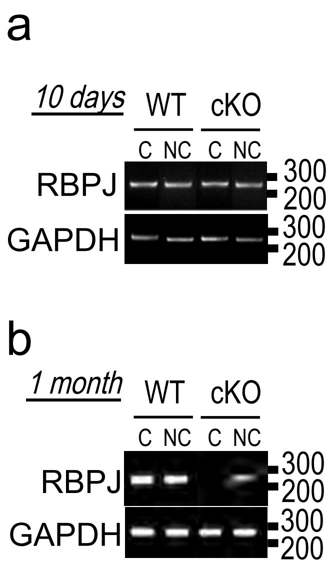

Supplementary Figure 3. Cardiomyocyte and postnatal specific deletion of RBPJ

a,b) Cardiomyocyte-specific inactivation of *Rbpj*. PCR analyses of *Rbpj* exons 6 and 7 in cardiomyocytes (C) and non-cardiomyocytes (NC) isolated from hearts of conditional knockout (cKO, *Myl*$2^{Cre/+}$, *Rbpj*$^{flox/flox}$) and WT (*Myl*$2^{+/+}$, *Rbpj*$^{flox/flox}$) littermates 10 days **(a)** and 1 month **(b)** after birth. *Rbpj* deletion occurred in 1 month old, but not in neonatal, hearts.

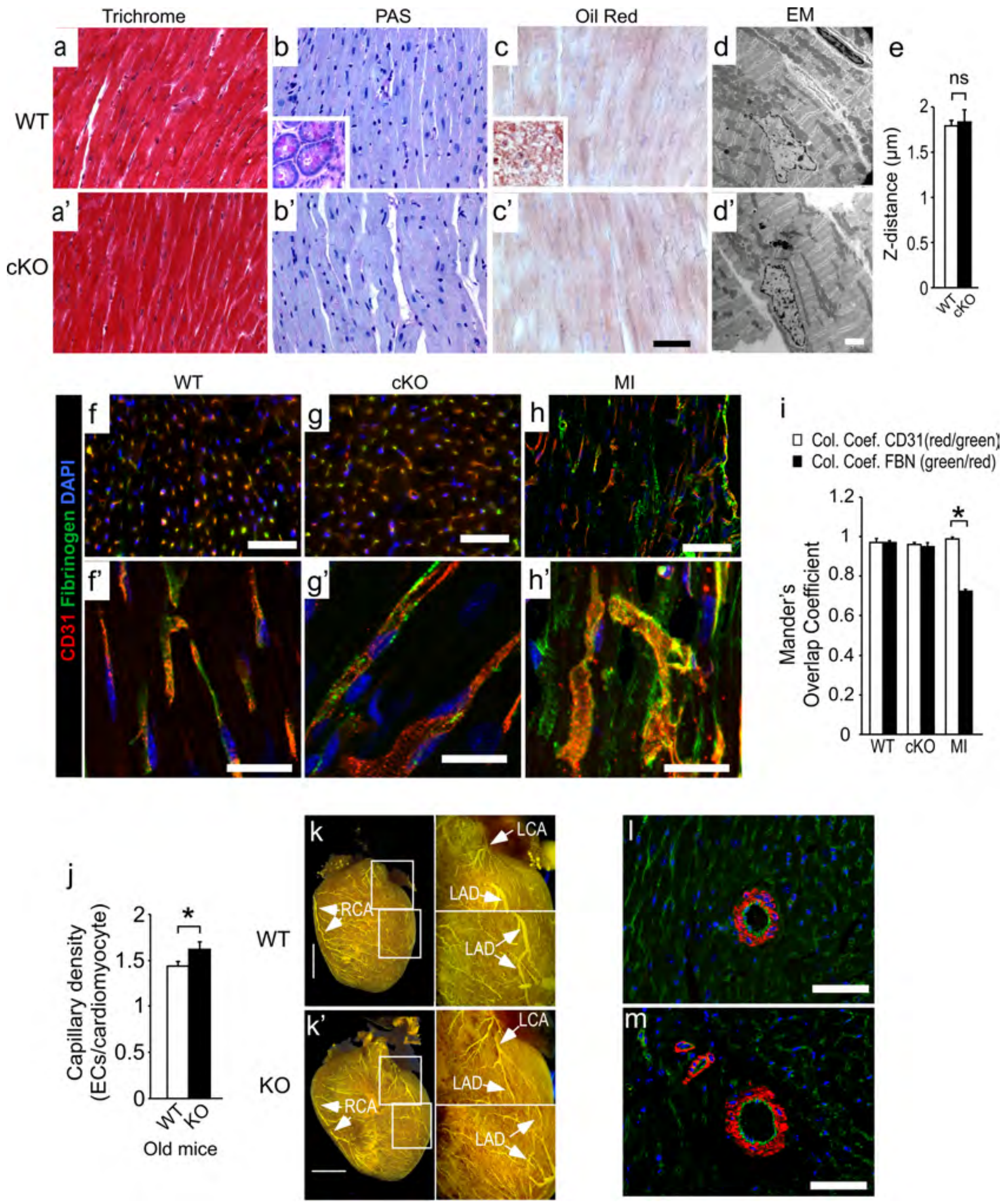

Supplementary Figure 4. Histological characterization, microvessel staining and coronary vasculature in RBPJ cKO versus WT hearts.

a-e) Histochemical staining of cKO (*Myl2*$^{Cre/+}$, *Rbpj*$^{flox/flox}$) and WT (*Myl2*$^{+/+}$, *Rbpj*$^{flox/flox}$) hearts with Masson's trichrome **(a,a')**, PAS (for glycogen accumulation) **(b,b')** and by Oil red-O (for lipid accumulation) **(c,c')** using mouse fat **(c inset)** and liver **(b inset)** as positive

controls. Transmission electron microscopy of a 2 year-old mouse heart from WT **(d)** and cKO **(d')** reveals normal myocardial structure and Z-line spacing, quantified in **e**, consistent with normal myocardial appearance. Scale bar 50μm (a-c'), 2μm (d, d')

f-i) Microvessel integrity assessed by fluorescent immunostaining of LV anterior wall histological sections. Fluorescence of fibrin/fibrinogen (green), CD31 (red), and DAPI (blue) from WT **(f, f')**, cKO **(g, g')** and WT MI **(h,h')**. Co-localization of fibrin/fibrinogen with CD31 was quantified using Mander's colocalization coefficient (maximum colocalization coefficient=1) **(i)**. A high degree of co-localization reflects the absence of fibrin/fibrinogen deposition outside vessels in the WT and cKO hearts, whereas leakiness was apparent by the lower Mander's coefficient of characteristically leaky vasculature in infarcted myocardium. Scale bars, 50μm **(f-h)** and 20μm **(f'-h')**. Error bars indicate s.e.m, n=3 mice (all cases). Asterisk, $P<0.05$.

j,k) Vessel analysis of aged (23-26 months old) mice. Quantification of microvessel density (ECs per cardiomyocyte) **(j)** by counting capillaries and cardiomyocytes stained with FITC-LEA and Alexa 568-phalloidin respectively. Error bars indicate s.e.m., n≥4. Asterisk, $P<0.05$. Coronary vessels of WT **(k)** and cKO **(k')** were identified in the whole heart by Microfil polymer infusion (Methods). RCA: Right Coronary Artery; LCA: Left Coronary Artery; LAD: Left Anterior Descending artery. Sclae bar 2mm.

l,m) Vessel analysis. Coronary vessels were identified on heart sections by smooth muscle staining (red) and endothelial cell (EC) CD31 (green) stain **(l, m).** Scale bars, 50μm.

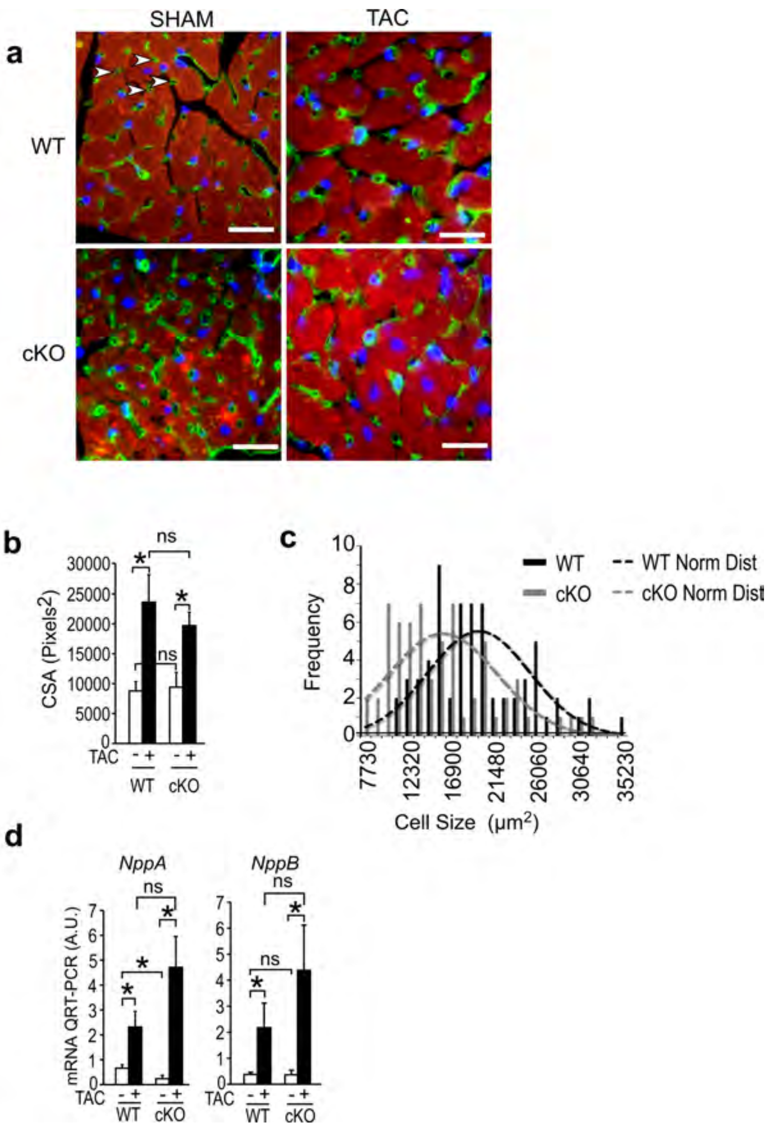

Supplementary Figure 5. Analysis of cardiomyocytes size and hypertrophic response in WT and RBPJ cKO mice.

a,b) Hypertrophy morphometric analysis of hearts from 3 month old cKO and WT littermates subjected to 14 days TAC (+) or sham operation (-). Cross sectional area (CSA) of LV anterior wall cardiomyocytes on histological section **(a)** show comparable values for WT and cKO hearts **(b)**. Sections **(a)** stain with phalloidin (red), isolectinB4 (green) and Dapi (blue). CSA, cross-sectional area. Error bars indicate s.e.m., n=4. Asterisk, $P<0.05$. Scale bar 20μm.

c) Size frequency distributions of isolated adult ventricular cardiomyocytes from WT and cKO genotypes. Length x width measurements were determined from primary cultures of adult cardiomyocytes of WT (n=65 cardiomyocytes) and cKO (n=64 cardiomyocytes) littermates prepared by the Langendorff perfusion method (see Methods). There was no statistical difference (p=00013, ANOVA, 1-tailed) and overlapping normal distributions (dashed lines) between the WT and cKO genotypes.

d) Gene expression analyses from 3 month-old cKO and WT littermates subjected to 14 days TAC (+) or sham operation (-). mRNA levels for *NppA* and *NppB* (encoding atrial and brain natriuretic peptides) by Q-RT-PCR (Supplementary Table 5), normalized to β-actin, are not induced at baseline, but upregulated by TAC in both genotypes. For *NppA*, n = 4,6,6,5; *NppB*, n=5,5,7,5 mice; respectively. Error bars indicate s.e.m. Asterisk, $P<0.05$; ns, not significant.

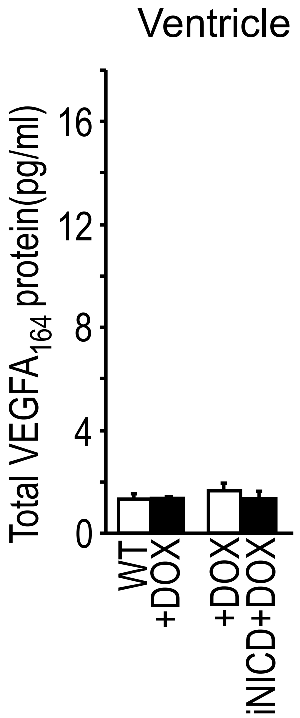

Supplementary Figure 6. Effect of doxycycline on VEGFA production.

$VEGFA_{165}$ protein quantification by ELISA on WT and iNICD heart tissue treated or untreated with doxycycline. No effect on VEGFA production is detected. Error bars indicate s.e.m, n=4 biological replicates in all instances. Asterisk, $P<0.05$.

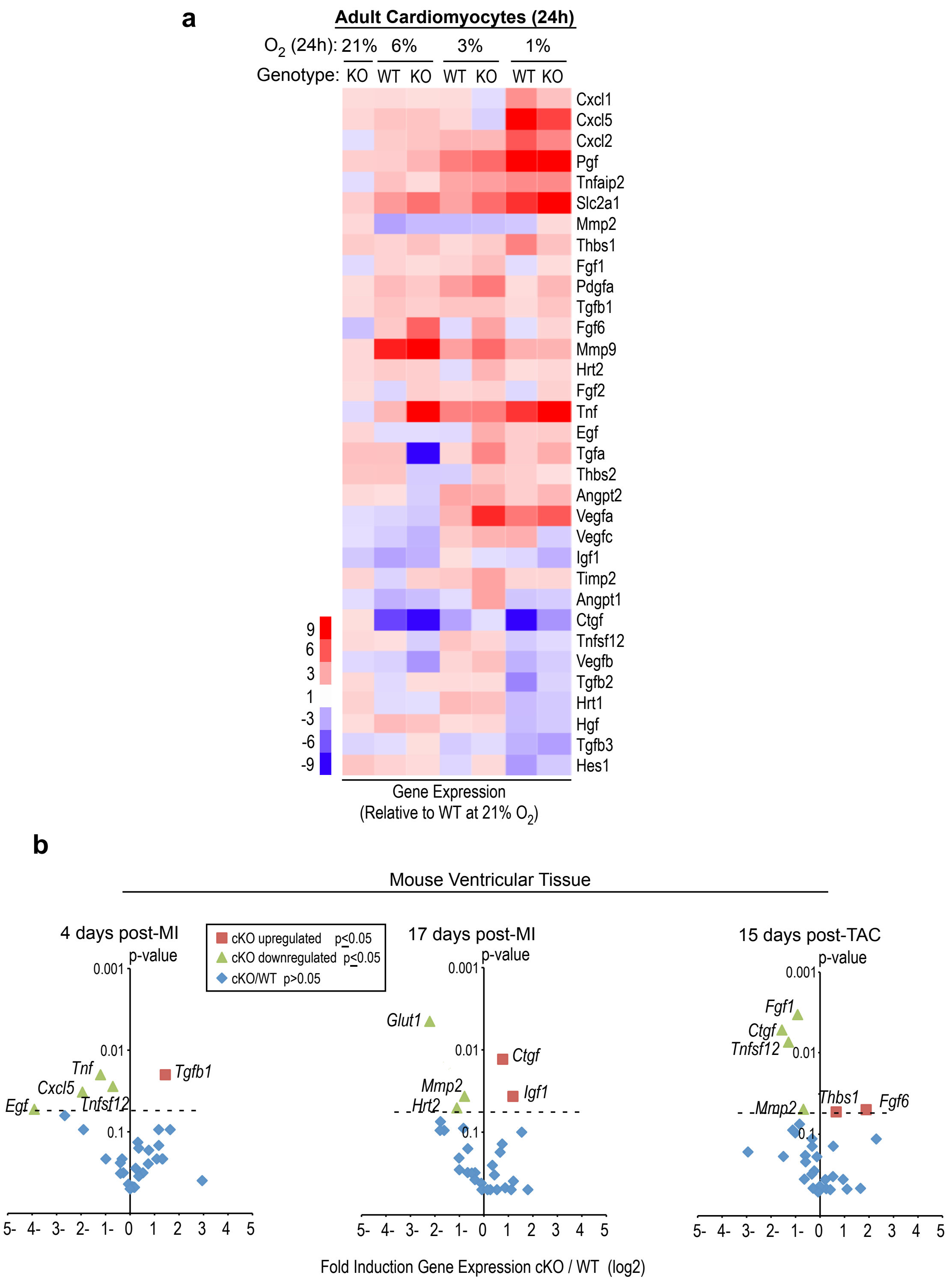

.

Supplementary Figure 7. Angiogenic factor gene expression heatmap from cKO and WT isolated adult cardiomyocytes at 21, 6, 3 and 1%O_2 and from adult heart tissue after TAC and MI.

a) Isolated adult cardiomyocytes prepared from cKO (*Myl*2$^{Cre/+}$, *Rbpj*$^{flox/flox}$) and WT (*Myl*2$^{+/+}$, *Rbpj*$^{flox/flox}$) mice were cultured for 24h at ambient oxygen (21%O_2) and 6%, 3% and 1% before being processed for Q-RT-PCR analysis (primer list on Supplementary Table 6). Expression of each gene was normalized to the level of *Actb*. Values in heatmap are further normalized to WT cardiomyocytes at 21% O_2 (Inset to left shows color scale) and hierarchically clustered by Cluster3.0 (Gene profiling is from n≥3 biological replicates shown with statistics in Fig. 4a).

b) Gene expression analysis of 29 secreted angiogenic factors, 3 Notch targets and in heart ventricular tissue and isolated adult ventricular cardiomyocytes from cKO (*Myl*2$^{Cre/+}$, *Rbpj*$^{flox/flox}$) and WT (*Myl*2$^{+/+}$, *Rbpj*$^{flox/flox}$) mice by Q-RT-PCR normalized to *ActB* (primers are listed in Supplementary Table 6). Mouse ventricular tissue was collected from untreated (Baseline, **Fig. 2l**) or stressed [15 days after transaortic constriction (TAC) or 4 days and 17 days post myocardial infarction (MI)] cKO and WT mice. Volcano plots portray the cKO to WT ratio (X-axis) relative to p-value (Mann-Whitney test). Red and green points indicate statistically significant ($P \leq 0.05$, n≥4) induction or repression of gene expression, respectively; blue points indicate no statistically significant difference.

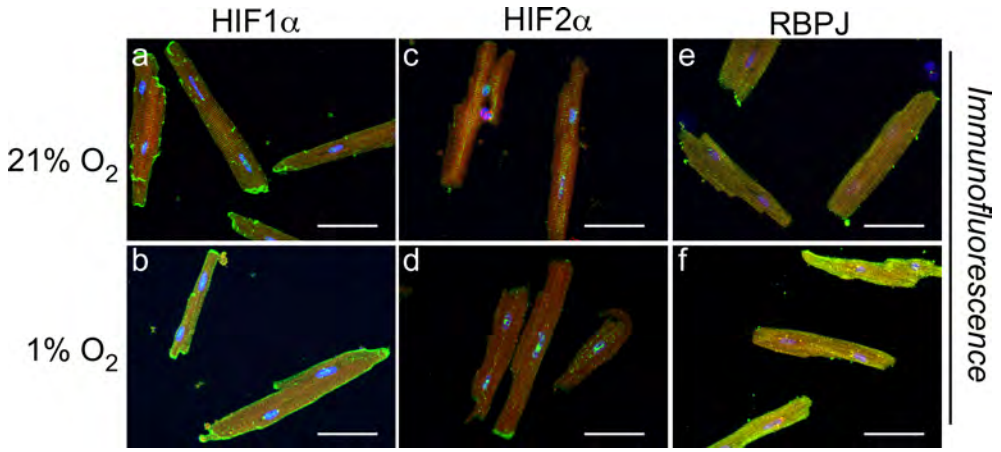

Supplementary Figure 8. HIF1α, HIF2α and RBPJ immunofluorescence in isolated adult cardiomyocytes.

a-f) Immunodetection of Hif1α **(a,b)**, Hif2α **(c,d)** and RBPJ **(e,f)** on isolated adult cardiomyocytes after 14 hours of cell culture under normoxic **(a, c, e)** and hypoxic **(b, d, f)** conditions. Hif1α, Hif2α and RBPJ staining represented in green, phalloidin in red and DAPI in blue. Pictures representative of more than 3 experiments. Scale bars, 50 µm.

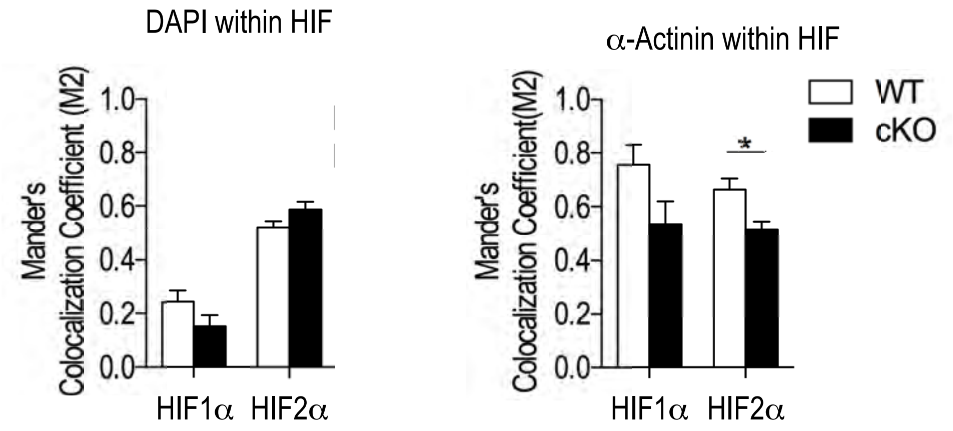

Supplementary Figure 9. HIF1α and HIF2α cytoplasmic Mander's colocalization coefficient.

Quantification of HIF1α and HIF2α immunostaining of cKO and WT heart sections at baseline **(from Fig. 4g)** co-stained with cardiac α-actinin (red) and DAPI (blue). Nuclear colocalization of DAPI within HIF1α or HIF2α **(left graph)** and cytoplasmic colocalization of α-actinin within HIF1α or HIF2α **(right graph)** was calculated using Mander's colocalization coefficient (M2). M1 values shown in **Fig. 4g**. Error bars indicate s.e.m., n=3 and 4 biological replicates for WT and cKO mice. Asterisk, $P<0.05$.

Fig. 4e

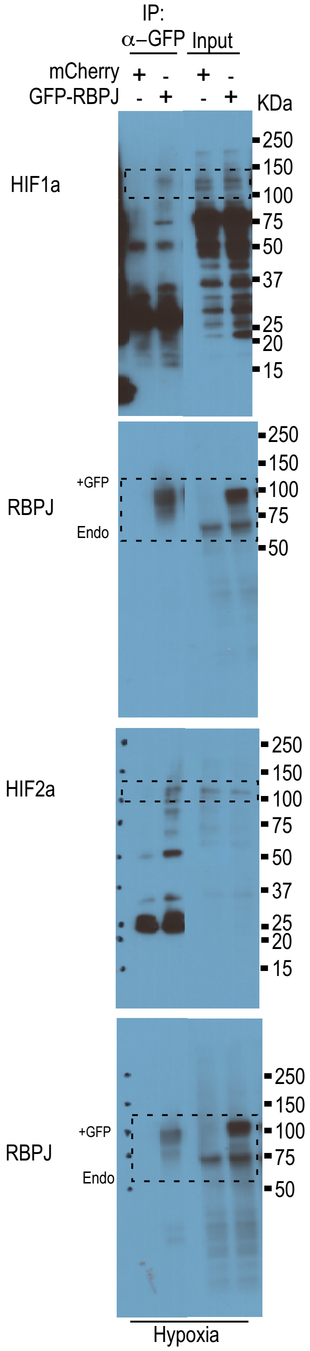

Supplementary Fig. 1b

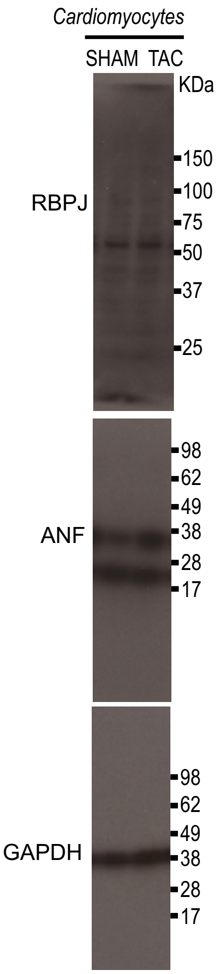

Supplementary Fig. 3

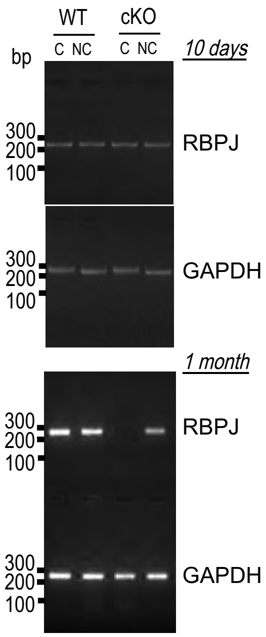

Supplementary Figure 10. Uncropped images of gel chromatography.

Raw images are shown for all display and supplementary figures as indicated.

SUPPLEMENTARY TABLES

Supplementary Table 1. Survival of *Myl2*$^{Cre/+}$, *Rbpj*$^{flox/flox}$ mouse line

Myl2$^{Cre/+}$, *Rbpj*$^{flox/flox}$ x *Myl2*$^{+/+}$, *Rbpj*$^{flox/flox}$

Cre/+; Flox/Flox	29 (22.8%)
Cre/+; Flox/+	35 (27.5%)
+/+; Flox/Flox	33 (25.9 %)
+/+; Flox/+	27 (21.2%)
Total	127 (100%)

Supplementary Table 2. Echocardiography measurements from WT and RBPJ cKO before and after 14 days of TAC

	WT	WT-TAC	cKO	cKO-TAC
IVSd (mm)	0.67 ± 0.08	0.78 ± 0.09	0.62 ± 0.02	0.72* ± 0.1
LVIDd (mm)	3.67 ± 0.33	4.40 ± 0.85	3.83 ± 0.57	4.41 ± 0.68
LVPWd (mm)	0.66 ± 0.08	0.82* ± 0.10	0.62 ± 0.04	0.72* + 0.1
IVSs (mm)	1.2 ± 0.16	1.24 ± 0.12	1.11 ± 0.06	1.14 ± 0.13
LVIDs (mm)	1.99 ± 0.28	2.98* ± 0.96	2.14 ± 0.58	3.07* ± 0.79
LVPWs (mm)	1.3 ± 0.13	1.31 ± 0.15	1.15 ± 0.18	1.20 ± 0.14
HR (bpm)	617.1 ± 59	470* ± 60	624 ± 62	538*# ± 69
Ao-ET (ms)	44.2 ± 4	56* ± 10	46 ± 4	51* ± 6
Ao-HR (bpm)	629 ± 74	488 ± 71	599 ± 68	539 ± 65
%FS	45.7 ± 5.9	33.6* ± 10.3	44.9 ± 7.1	31.2* ± 7.5
EDD/PWD	5.6 ± 0.56	5.54 ± 1.56	6.2 ± 1	6.32 ± 1.82
VCF (circ/s)	10.38 ± 1.57	6.24* ± 2.47	9.94 ± 1.7	6.19* ± 1.66
LVDd/BW	0.10 ± 0.01	0.14* ± 0.04	0.12 ± 0.01	0.143* ± 0.021
LVM (d) (mg)	81.9 ± 26.5	137* ± 34.5	22.4 ± 22.4	119.5* ± 24.4
P.P.		181.6 ± 29.1		192.6 ± 29.3
D.P.		119.9 ± 36.2		116.5 ± 31.9
Gradience		61.6 ± 10.7		76.1 ± 46.7
∆Mass		1.1 ± 3.9		1.2

*, p<0.05 WT vs WT-TAC and cKO vs cKO-TAC
#, p<0.05 WT-TAC vs cKO-TAC
n= 7, 7, 10 and 9 mice for WT, WT TAC, cKO and cKO TAC, respectively

Supplementary Table 3: *Vegfa* promoter analysis primers and RBPJ and HIF predicted binding sites by whole genome rVista precomputed analysis

TABLE 3a

GENE	FORWARD (5'-3')	REVERSE (5'-3')
Vegfa	ATCGCGTGCAGTATATG	GCCATAAAACAACGACCT

TABLE 3b

	RBPJ	HIF
Vegfa	160,243,**1422,1478,1522,1541**, 2040,2116,4794	**1541,1542**

Primers used for genomic ChIP PCR analysis on Roche LightCyler 2.0. The primer pairs (a) were at positions -1403 and -1561 relative to the start site of transcription, and spanned predicted RBPJ consensus binding sites located at positions -1422, -1478, -1522, 1541 and HIF predicted binding sites 1541, 1542 (b).

Supplementary Table 4. Hemodynamic parameters

Genotype at 21% O_2:	WT^{Myl2}	cKO^{Myl2}	WT^{Myh6}	$icKO^{Myh6}$
Cardiac Output (ml.min)	12.2±0.3	12.1±0.1	13.0±0.3	12.3±0.1
Stroke Volume (μl)	22.3±0.5	21.2±0.2	22.6±0.7	22.6±0.2
Heart Rate (bpm)	561.1±23.2	580.0±6.6	579.4±23.6	541.6±8.0
MAP (mmHg)	120.5±2.2	122.8±2.0	119.8±1.7	119.0±1.8
VR (mmHg.min/ml)	9.9±0.2	10.4±0.6	9.2±0.2	9.8±0.4
Delivery O_2 (ml O_2/min)	2.2±0.1	2.1±0.0	2.3±0.0	2.2±0.0

Measurements taken at baseline (21%O_2) showing no significant differences between cKO and icKO compared to their respective controls WT^{Myl2} and WT^{Myh6}. Values are means ± SEM. MAP, Mean arterial pressure; VR, vascular resistance. n=4 for each group.

Supplementary Table 5. Primers used for RT PCR gene expression analysis on Roche LightCycler 2.0

GENE	FORWARD (5'-3')	REVERSE (5'-3')	REF. SEQ.
Rbpj	GAATTTCCACGCCAGTTCAC	ATACAGGGTCGTCTGCATCC	NM_001080927.1
NppA	TTGGAGCAAATCCTGTGTAC	CTTCCTCAGTCTGCTCACTC	NM_008725.2
NppB	AAGAGTCCTTCGGTCTCAAG	CCAGGAGGTCTTCCTACACC	NM_008726.4

Supplementary Table 6. Primers used for RT PCR gene expression analysis on the Applied Biosystem 7900HT with Biorad iQ SYBR Green Supermix in 384-well plates

GENE	FORWARD (5'-3')	REVERSE (5'-3')	REF. SEQ.
Angpt1	TGCACTAAAGAAGGTGTTTTGCT	TGCACAGTCTCGAAATGGTTT	NM_009640
Angpt2	GGAGACCGTCAACAGCTTG	CTTCTTTACGGATAGCAACCGAG	NM_007426
Ctgf	GACCCAACTATGATGCGAGCC	TCCCACAGGTCTTAGAACAGG	NM_010217
Cxcl1	ACTGCACCCAAACCGAAGTC	TGGGGACACCTTTTAGCATCTT	NM_008176
Cxcl2	CCAACCACCAGGCTACAGG	GCGTCACACTCAAGCTCTG	NM_009140
Cxcl5	ATGGCGCCGCTGGCATTTCT	CGCAGCTCCGTTGCGGCTAT	NM_009141
Egf	AGAGCATCTCTCGGATTGACC	CCCGTTAAGGAAAACTCTTAGCA	NM_010113
Fgf1	CAGCTCAGTGCGGAAAGTG	TGTCTGCGAGCCGTATAAAAG	NM_010197
Fgf2	GCGACCCACACGTCAAACTA	TCCCTTGATAGACACAACTCCTC	NM_008006
Fgf6	CAGGCTCTCGTCTTCTTAGGC	TTCACACCCGAAATCTCTCCA	NM_010204
Hgf	ACTTCTGCCGGTCCTGTTG	GGGATGGCGACATGAAGCA	NM_010427
Igf1	CACATCATGTCGTCTTCACACC	GGAAGCAACACTCATCCACAATG	NM_00111127 4
Mmp2	CCTGGACCCTGAAACCGTG	TCCCCATCATGGATTCGAGAA	NM_008610
Mmp9	GCAGAGGCATACTTGTACCG	TGATGTTATGATGGTCCCACTTG	NM_013599
Pdgfa	TGTGCCCATTCGCAGGAAG	GAGGTATCTCGTAAATGACCGTC	NM_008808
Pgf	AGTGGAAGTGGTGCCTTTCAA	GTGAGACACCTCATCAGGGTA	NM_009640
Tgfa	TCTGGGTACGTGGGTGTTC	ACAGGTGATAATGAGGACAGCC	NM_007426
Tgfb1	AGCTGGTGAAACGGAAGCG	GCGAGCCTTAGTTTGGACAGG	NM_010217
Tgfb2	AGAATCGTCCGCTTTGATGTC	TCTGGTTTTCACAACCTTGCT	NM_008176
Tgfb3	GGACTTCGGCCACATCAAGAA	TAGGGGACGTGGGTCATCAC	NM_009140
Thbs1	CCTGCCAGGGAAGCAACAA	ACAGTCTATGTAGAGTTGAGCCC	NM_009141

Thbs2	CTGGGCATAGGGCCAAGAG	GTCTTCCGGTTAATGTTGCTGAT	NM_010113
Timp2	GCAACCCCATCAAGAGGATTC	GGGGCCGTGTAGATAAACTCG	NM_010197
Tnf	CAGGCGGTGCCTATGTCTC	CGATCACCCCGAAGTTCAGTAG	NM_008006
Tnfaip2	GGAGGTGGCAGCGGAACGTC	AAGGCGCGCTGGTAGCTCCTC	NM_010204
Tnfsf12	CCGCCAGATTGGGGAATTTAC	AGTCCAAAGTAGGTTAGGAAGGG	NM_010427
Vegfa	CTTGTTCAGAGCGGAGAAAGC	ACATCTGCAAGTACGTTCGTT	NM_001111274
Vegfb	GCCAGACAGGGTTGCCATAC	GGAGTGGGATGGATGATGTCAG	NM_008610
Vegfc	GTGAGGTGTGTATAGATGTGGGG	ACGTCTTGCTGAGGTAACCTG	NM_013599
ActinB	GTGACGTTGACATCCGTAAAGA	GCCGGACTCATCGTACTCC	NM_008808
Slca1	GCAGTTCGGCTATAACACTGG	GCGGTGGTTCCATGTTTGATTG	NM_008827
Hrt1	CCGACGAGACCGAATCAATAAC	TCAGGTGATCCACAGTCATCTG	NM_031199
Hrt2	AAGCGCCCTTGTGAGGAAAC	TCCCCACGTCGATGGTCTC	NM_011577
Hes1	TCAACACGACACCGGACAAAC	ATGCCGGGAGCTATCTTTCTT	NM_009367
